# A substitution mutation in cardiac ubiquitin ligase, FBXO32, is associated with an autosomal recessive form of dilated cardiomyopathy

**DOI:** 10.1186/s12881-016-0267-5

**Published:** 2016-01-14

**Authors:** Zuhair N. Al-Hassnan, Zarghuna MA. Shinwari, Salma M. Wakil, Sahar Tulbah, Shamayel Mohammed, Zuhair Rahbeeni, Mohammed Alghamdi, Monther Rababh, Dilek Colak, Namik Kaya, Majid Al-Fayyadh, Jehad Alburaiki

**Affiliations:** Cardiovascular Genetics Program, King Faisal Specialist Hospital & Research Centre, Riyadh, Saudi Arabia; Deptartment of Medical Genetics, King Faisal Specialist Hospital & Research Centre, Riyadh, Saudi Arabia; Department of Genetics, King Faisal Specialist Hospital & Research Centre, Riyadh, Saudi Arabia; Department of Pathology and Laboratory Medicine, King Faisal Specialist Hospital & Research Centre, Riyadh, Saudi Arabia; Department of Biostatistics and Scientific Computing, King Faisal Specialist Hospital & Research Centre, Riyadh, Saudi Arabia; Heart Center, King Faisal Specialist Hospital & Research Centre, Riyadh, Saudi Arabia; College of Medicine, Alfaisal University, Riyadh, Saudi Arabia; Department of Medical Genetics, MBC-75, King Faisal Specialist Hospital & Research Centre, Takhassusi Street, PO Box 3354, Riyadh, 11211 Saudi Arabia

**Keywords:** Cardiomyopathy, *FBXO32*, Ubiquitin proteasome system

## Abstract

**Background:**

Familial dilated cardiomyopathy (DCM) is genetically heterogeneous. Mutations in more than 40 genes have been identified in familial cases, mostly inherited in an autosomal dominant pattern. DCM due to recessive mutations is rarely observed. In consanguineous families, homozygosity mapping and whole exome sequencing (WES) can be utilized to identify the genetic defects in recessively inherited DCM.

**Methods:**

In a consanguineous family with four affected siblings with severe DCM, we combined homozygosity mapping, linkage analysis and WES, to uncover the genetic defect.

**Results:**

A region of homozygosity (ROH) on chromosome 8q24.13–24.23 was found to be shared by all of the four affected siblings. WES detected ~47,000 variants that were filtered to a homozygous mutation (p.Gly243Arg) in the *FBXO32* gene, located within the identified ROH. The mutation segregated with the phenotype, replaced a highly-conserved amino acid, and was not detected in 1986 ethnically-matched chromosomes. *FBXO32,* which encodes a muscle-specific ubiquitin ligase, has been implicated in the pathogenesis of cardiomyopathy through the ubiquitin proteasome system (UPS). In addition, *FBXO32*-knockout mice manifest with cardiomyopathy. Screening the index patient for all of the WES variants in 48 genes known to be implicated in hypertrophic and dilated cardiomyopathy was negative.

**Conclusions:**

Our data suggest that *FBXO32* is a candidate gene for recessive DCM. Acting as a cardiac ubiquitin ligase, mutated *FBXO32* could perturb the degradation of target proteins in the UPS, the impairment of which has been observed in cardiomyopathy. Our work proposes that genes encoding other ubiquitin ligases could also be implicated in familial cardiomyopathy.

**Electronic supplementary material:**

The online version of this article (doi:10.1186/s12881-016-0267-5) contains supplementary material, which is available to authorized users.

## Background

Dilated cardiomyopathy (DCM; OMIM 115200), characterized by impaired systolic function and left ventricular enlargement, is considered to be the most common indication for cardiac transplantation [1, 2]. A familial pattern, mostly inherited as autosomal dominant, has been observed in about 20–35 % of DCM cases with remarkable genetic heterogeneity; disease-associated mutations have been identified in more than 40 genes [3, 4]. A recessively inherited form of familial DCM is rarely observed and is often associated with extra-cardiac manifestations such as skeletal myopathy, hypotonia, hepatic encephalopathy, and impaired fatty acid oxidation defects.

The ubiquitin proteasome system (UPS) is crucial for the regulation of cellular protein degradation. The target proteins for degradation undergo an initial step of polyubiquitination before their destruction by the 26S proteasome [5]. The UPS cascade consists of three components: a ubiquitin-activating enzyme (E1), a ubiquitin-conjugating enzyme (E2), and a ubiquitin-ligase enzyme (E3), which is the substrate-specific component of the UPS. Impaired UPS has recently been implicated in the pathogenesis of heart failure, cardiac ischemia, and cardiomyopathy (CMP) [6, 7]. The protein encoded by *FBXO32*, also known as atrogin-1 and MAFbx, is an E3 ligase that is expressed selectively in skeletal muscles and cardiomyocytes and plays a critical role in muscle atrophy [8, 9], as well as in the development of cardiac hypertrophy and atrophy [10, 11]. In support of an association between deficient FBXO32 protein and CMP, a recent study has shown that atrogin-1 knockout mice develop CMP with intracellular protein accumulation and cardiomyocyte apoptosis [12].

## Methods

### Subjects with cardiomyopathy

**Fig. 1:**
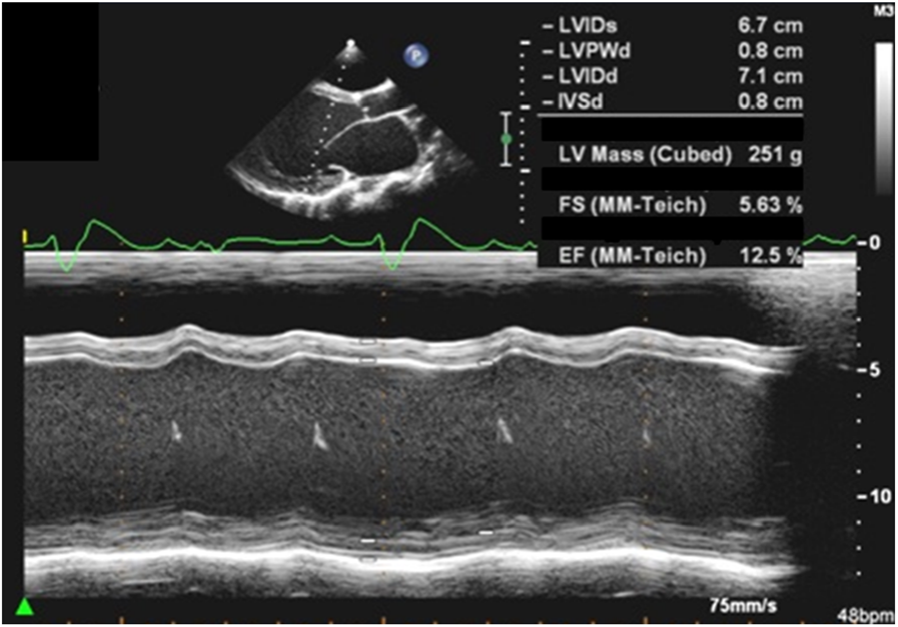
M-mode echocardiographic recordings from the index patient showing severe dilatation of the left ventricle (LVIDs: Left ventricle internal diameter during systole; LVIDd: Left ventricle internal diameter in diastole; both are markedly increased) and poor contractility with an ejection fraction (EF) of 12.5 %

**Fig. 2:**
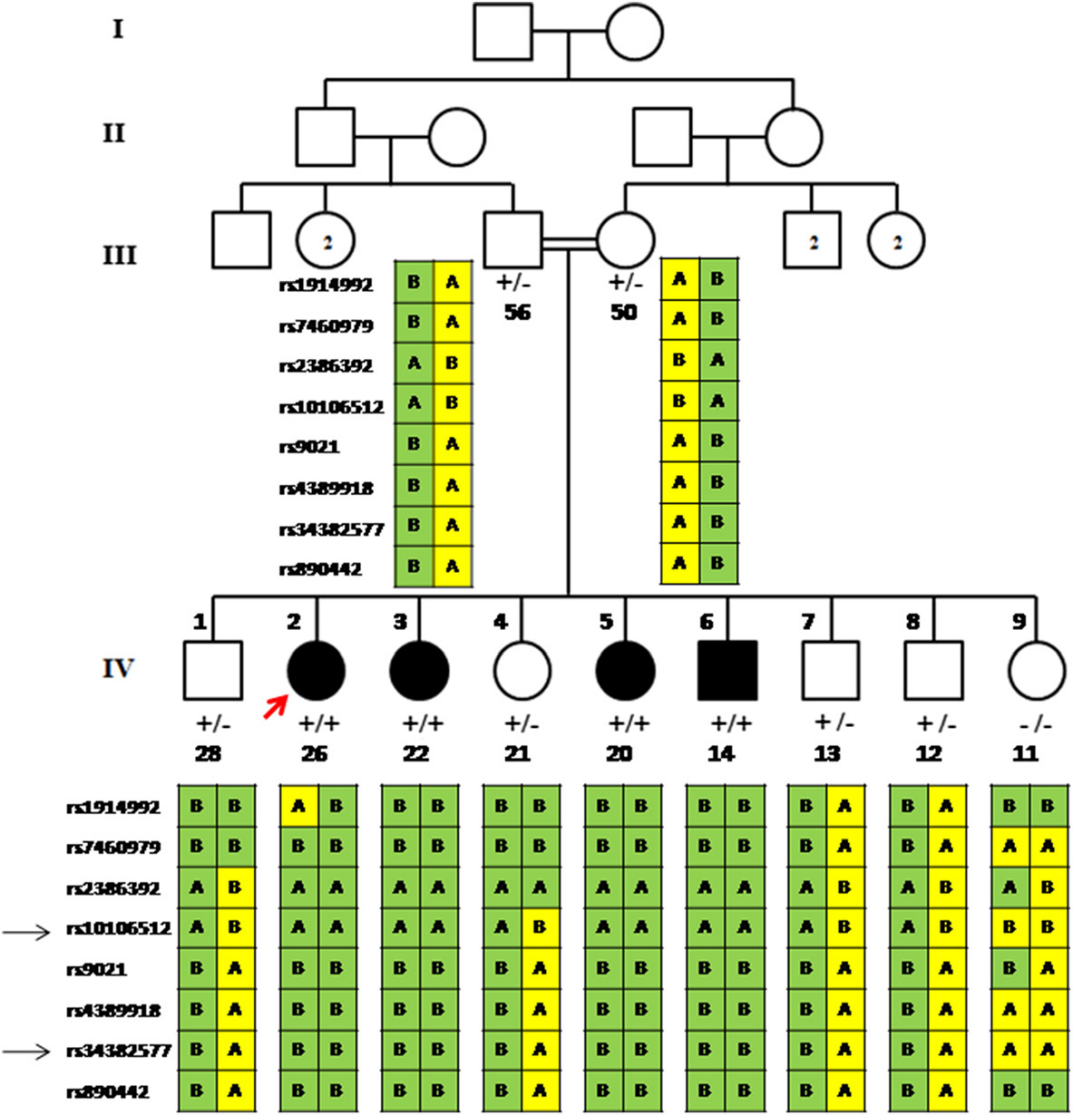
Family pedigree showing the four affected siblings (filled symbols). Haplotypes spanning 8q24.12–q24.21 are shown under each symbol with two black arrows indicating the shared homozygous region between the affected individuals. The presence of the mutant allele (c.727G > C) is indicated by (+). The index case is indicated by the red arrow. The number below the symbol represents the age (in years). Echocardiogram was performed on the parents of the index case and all of their offspring

The index patient was a 26-year-old female who presented with a history of progressive shortness of breath and easy fatigability for 2 months. On examination, her vital signs revealed a blood pressure of 95/60 and a pulse rate of 95 beats per minute. The jugular venous pressure was 4 cm above the sternal angle. A heart examination revealed normal first and second heart sounds with blowing systolic murmur at the apex. The liver was palpable. There were no ascites or lower limb edema. A chest X-ray revealed cardiomegaly and pulmonary edema, and a 12-lead electrocardiogram showed sinus tachycardia with bifascicular block. An echocardiogram revealed a dilated left ventricle with severe global hypokinesia and poor left ventricular systolic function (Fig. 1). Despite maximal medical therapy, she developed decompensated heart failure, for which she underwent successful orthotropic heart transplantation. Family screening with transthoracic echocardiograms revealed three affected siblings: two asymptomatic sisters and one 14-year-old brother who had a history of dyspnea and orthopnea but had never been evaluated (Fig. 2, Table 1). Their parents and the other five siblings had normal echocardiograms. None of the affected patients had skeletal muscle weakness or abnormal creatine kinase levels. The subjects were enrolled in the study after obtaining written informed consent. This project was approved by the Research Advisory Council at KFSH&RC.

**Table 1:** Echocardiographic findings of the four affected individuals. EF: ejection fraction; FS: fractional shortening; LV: left ventricle; LVDd: left ventricular diastolic diameter; MR: mitral regurgitation; RV: right ventricle; TR: tricuspid regurgitation; yrs: years

Individual	Age (yrs)	Echocardiogram	EF	LVDd	FS
IV:2	26	severely dilated LV. severely reduced LV systolic function. moderate MR.	12.5 %	63	7.8 %
IV:3	22	moderately dilated LV. severely reduced LV systolic function. trace MR.	25 %	57	15.8 %
IV:5	20	moderately dilated LV. severely reduced LV systolic function. moderate MR.	25 %	59	16.9 %
IV:6	14	severely dilated LV and RV. severely reduced LV and RV systolic function. severe MR and TR	15 %	71	5.6 %

### Homozygosity mapping and linkage analysis

A recessive pattern of inheritance was assumed, because the family was consanguineous, the parents had unremarkable cardiac evaluations, and the disease affected both sexes. Genomic DNA was extracted from whole-blood samples obtained from all affected patients, their parents, and unaffected siblings. SNP-based genotyping was performed using AxiomTM CEU Human Array from AffymetrixR (Santa Clara, CA, USA). To detect regions of homozygosity (ROHs), SNPs were analyzed using AutoSNPa software [13]. Conventionally, ROH are defined as fragments where SNPs are homozygous for a stretch of consecutive alleles in affected individuals, and heterozygous, or homozygous for the other alleles, in unaffected members of the same family. Multipoint parametric linkage analysis was performed using Gene Hunter Easy Linkage Analysis Software module 4.0. A fully penetrant recessive model of inheritance was used, with a population disease allele frequency of 0.0001 and using Asian SNP allele frequencies.

### Whole exome sequencing

**Fig. 3:**
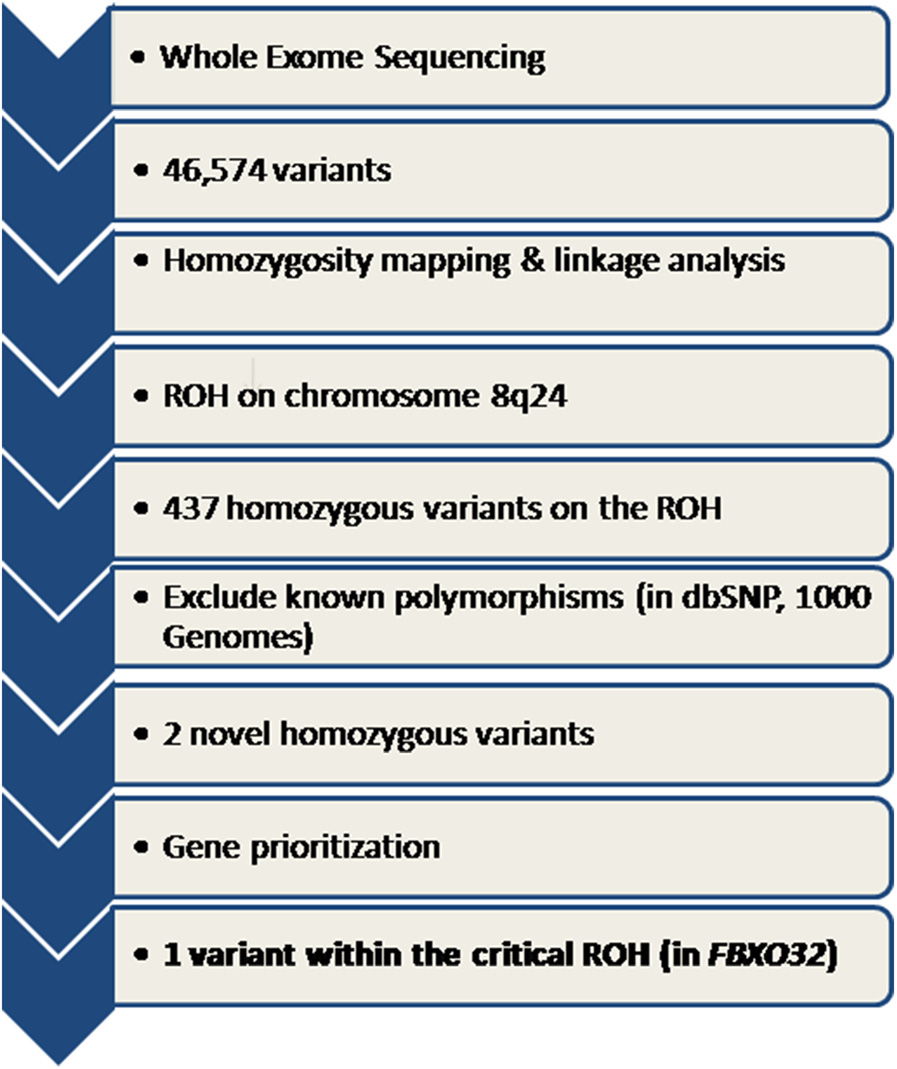
Flowchart showing the several steps of filtering the variants identified by whole exome sequencing. ROH: region of homozygosity

Whole exome sequencing (WES) was performed on the Illumina HiSeq 2000 platform. Samples were prepared and enrichment was carried out according to Agilent's SureSelect Protocols. Reads were mapped to the most recent build of the human genome (hg19/b37) using the Burrows-Wheeler Aligner package, version 0.6.2. Single nucleotide polymorphisms (SNPs) and insertion/deletion variants were called using the GATK Unified Genotyper for each sample. Variants detected by WES were filtered by restricting the analysis to homozygous changes within the ROH, followed by excluding previously reported polymorphisms (Fig. 3).

### Candidate gene search and mutation screening

Using the UCSC and Ensembl genome browsers [14, 15], the OMIM database [4], and the GeneDistiller2 software [16], candidate genes were analyzed based on their function, expression, biological pathways, or animal models. Genomic DNA of the affected patients, their parents, and siblings was amplified by PCR using intronic primers that were designed to flank (50–100 bp) the coding exons of the identified gene, as defined by the Ensembl Genome Browser [15]. PCR was performed in a final volume of 20 μl containing approximately 10 ng of genomic DNA, using standard conditions (primer sequences and conditions are available on request). Purified PCR amplicons covering the entire coding region of the selected genes were directly sequenced with the dideoxy chain-termination method using an ABI Prism Big Dye Terminator v3.1 Cycle Sequencing Kit, following the manufacturer’s instructions, and processed on an ABI 3730XL capillary sequencer (Applied Biosystems, CA, USA). Sequence analysis was performed using the SeqMan 6.1 module of the Lasergene (DNA Star Inc. WI, USA) software package, then compared with the reference GenBank sequence. Numbering commenced with the A of the ATG initiation codon as +1.

### Histopathology and immunohistochemistry

Histopathological and immunohistochemical studies were performed on ventricular myocardial tissue from the explanted heart of the proband, and on a control myocardial specimen from a non-cardiomyopathy case. To assess FBXO32 expression, a mouse monoclonal anti-human FBXO32 antibody raised against amino acids 1–300 (Santa Cruz Biotechnology, Dallas, Texas) was used for immunohistochemical staining. Antibody dilution was performed according to the manufacturer’s protocol.

## Results

### Homozygosity mapping and linkage analysis

**Fig. 4:**
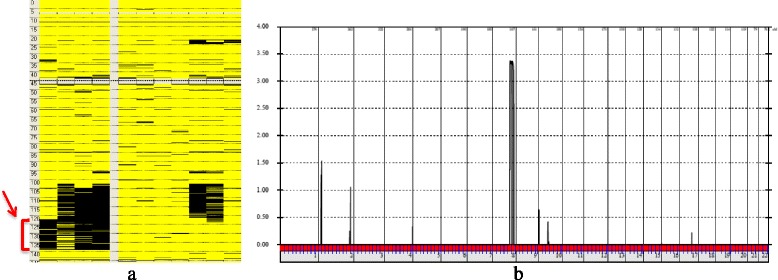
**a** Analysis of the SNP data using AutoSNPa software identified a block of homozygosity (arrow) of about 17 Mb on chromosome 8q24.13–q24.23 (8:122,185,539-139,111,674) that was shared by all of the four affected siblings (left) and not found in their parents and unaffected siblings (right). The horizontal axis indicates the chromosome numbers. The vertical axis indicates the LOD score. **b** GeneHunter Easy Linkage analysis showing the identified locus on 8q24 with a maximum LOD score of 3.37. Homozygous variants are displayed in black. Heterozygous variants are displayed in yellow

Homozygosity analysis identified a ROH (~17 Mb) on chromosome 8q24.13–q24.23 (8:122,185,539-139,111,674) that was shared by all of the four affected siblings (Fig. 4a). This single ROH, which has 111 genes, was not shared by their parents and the unaffected five siblings, and was the only ROH across the genome that was found to be shared by all of the affected siblings. Further analysis of the SNPs within the detected ROH narrowed the critical region to rs17288269 at 8:123,924,246 and rs13261097 at 8:125,185,945, which has 29 genes. All the SNPs within this narrowed region were homozygous in all the four affected siblings, except for one SNP (rs17256536 at 8:124,768,356) in individual IV-3 and another SNP (rs1080109 at 8:124,780,391) in individual IV-5. Linkage analysis verified this region with a maximum LOD score of 3.37 for the two markers rs16898165 and rs28522984, which are located at 8:124,232,985 and 8:124,351,429 (Fig. 4b).

### Whole exome sequencing

**Fig. 5:**
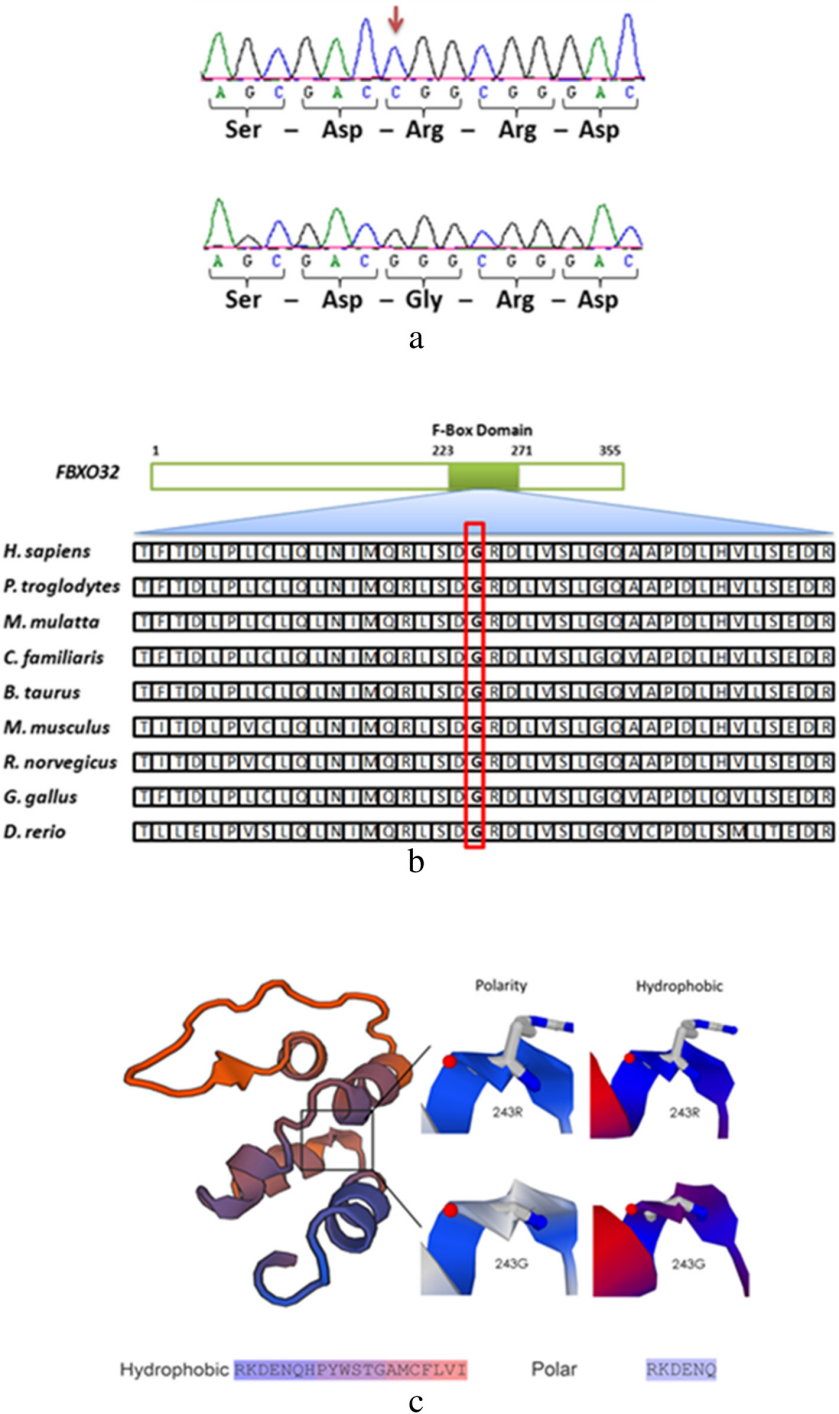
**a** The homozygous mutation (c.727G > C, p.G243R) is indicated by the arrow in the upper panel. The normal allele is shown in the lower panel. **b** Protein sequence alignment of *FBXO32* orthologs demonstrating that the glycine residue is a conserved amino acid down to zebrafish (*D. rerio*). **c** In Silico Protein model generated using Expasy-based modeling tools. On the left, the color of the model is based on the size properties of the amino acids. The color-coded polarity and hydrophobicity for the variant is given below the figure. The variant is predicted to destabilize the structure

WES of the index case identified 46,574 variants overlapping genes. Several steps to filter the variants were implemented (Fig. 3). The results of linkage analysis and homozygosity mapping were useful in restricting the analysis to the single ROH that had been identified to be shared by all the affected individuals. The extended ROH on chromosome 8q24 was found to have 437 homozygous variants across the 17 Mb region. These variants were further filtered by excluding reported polymorphisms in the dbSNP and 1000 Genomes databases, resulting in only two homozygous variants; p.His356Tyr in *OC90* and p.Gly243Arg in *FBXO32*. The *OC90* variant was excluded, as it is unlikely to be implicated in CMP. The mouse ortholog of *OC90* has been shown to be specifically expressed in the developing otocyst and to be involved in sensing orientation relative to gravity [17]. In addition, mice homozygous for a null *Oc90* allele exhibit altered otoliths and thin cupula, saccule, utricle and tectorial membranes, and have no cardiac phenotype [18]. The other variant identified, in *FBXO32* (8:124,510,127-124,553,493), is more likely to be associated with the cardiac phenotype in the family. In all of the four affected siblings, a homozygous c.727G > C mutation in the F-box domain was detected, resulting in the substitution of glycine with arginine at amino acid position 243 (p.Gly243Arg, Fig. 5a). This missense mutation segregated with the disease phenotype; the parents and four of the unaffected siblings were heterozygous carriers while one unaffected sibling was homozygous for the normal allele (Fig. 2). The mutation was not found in 1986 chromosomes from ethnically-matched normal controls.

We have also screened all the WES variants that overlap 48 OMIM genes implicated in dilated and hypertrophic familial CMP (Additional file 1: Table S1). All of the identified 62 variants have been previously reported in the dbSNP and/or 1000 Genomes databases as polymorphisms, except for two intronic variants in the *TTN* and *SGCD* genes.

### Bioinformatics-based prediction analysis for functional consequences of p.Gly243Arg

Several bioinformatics tools (Polyphen2 [19], PANTHER [20], SNPs&GO [21], and I-Mutant [22]) were utilized to predict the functional consequences of p.Gly243Arg (c.727G > C). The Polyphen2 program predicted the variant to be probably damaging. According to the PANTHER cSNP estimation, the change has a deleterious effect on protein function with a score of −3.624. SNPs&GO analysis predicted the p.Gly243Arg change as “disease” with a reliability score of 1, while I-Mutant predicted that the change will reduce the stability of the protein with a score of −0.67 (the “minus” score indicates reduced stability). Protein sequence alignment of *FBXO32* orthologs demonstrated that the glycine residue is a conserved amino acid down to zebrafish [14], suggesting that this amino acid has an essential function (Fig. 5b). We also modeled p.Gly243Arg of *FBXO32* on a template structure adapted from 2ovq (Fig. 5c). The analysis suggested that the replacement of the small and more hydrophobic glycine with the large, polar and less hydrophobic arginine may not only hinder normal protein folding, but also destabilize the protein, as was also predicted by the I-Mutant algorithm.

### Histopathology and immunohistochemistry

**Fig. 6:**
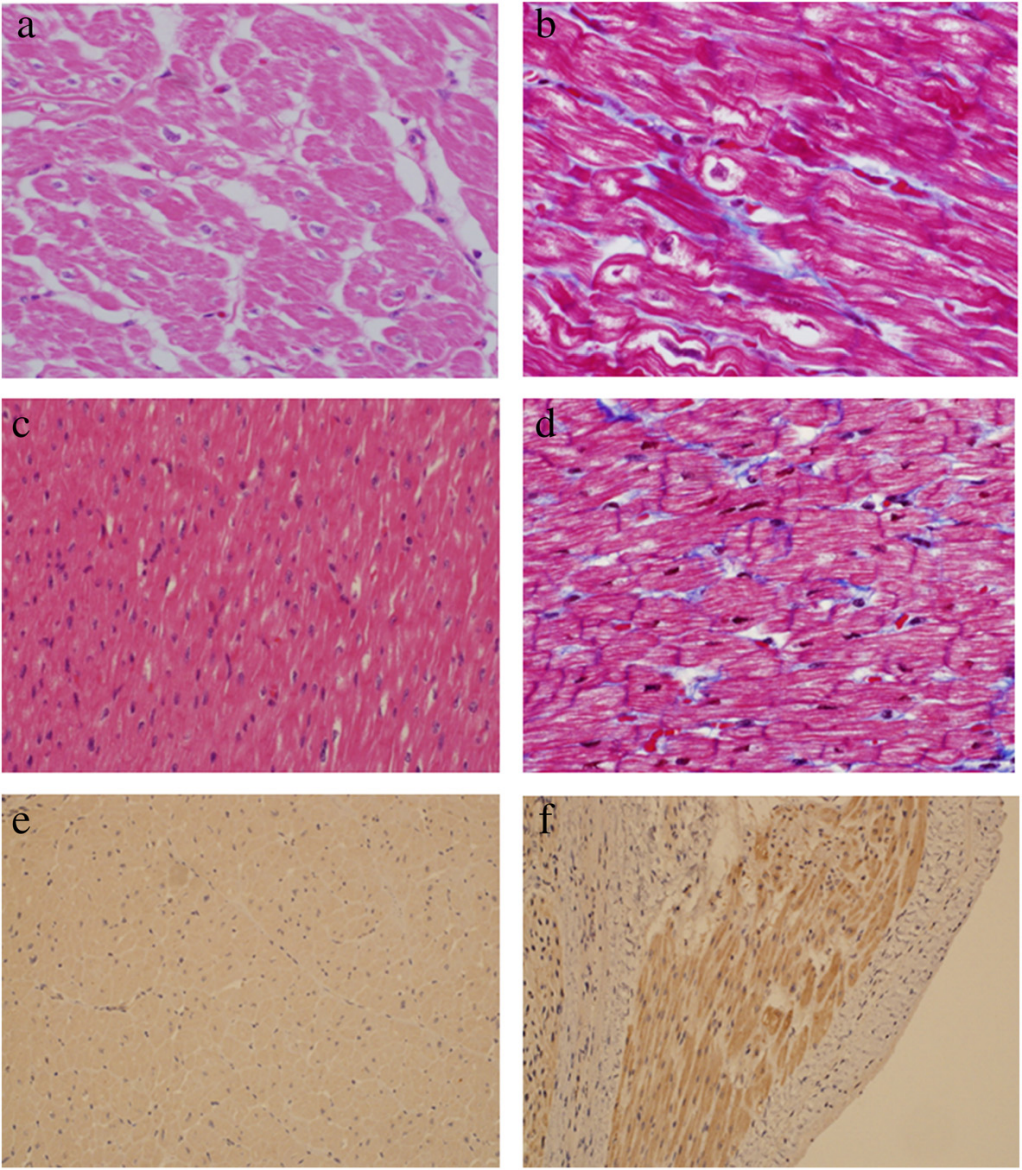
**a** Hematoxylin and eosin (H&E) and (**b**) trichrome staining of a left ventricular myocardial biopsy from the explanted heart of the proband showing hypertrophied myocytes, often with bizarrely shaped hyperchromatic nuclei and waving of the myocytes. **c** H&E and (**d**) trichrome staining from the control myocardium showing no cardiomyopathic changes, disarray, or fibrosis. **e** and (**f**) Immunohistochemical analysis of FBXO32 showing cytoplasmic staining with reduced protein expression in a section from the left ventricle of the proband (**e**) in comparison to a control myocardial specimen (**f**)

Histopathological analysis of the left ventricular myocardial biopsy from the explanted heart of the proband showed hypertrophied myocytes, often with bizarrely shaped hyperchromatic nuclei and waving of the myocytes (Fig. 6a and b). The immunohistochemical analysis of the FBXO32 protein showed cytoplasmic staining with reduced expression in the left ventricle of the index case in comparison to a control myocardial specimen from a non-cardiomyopathy case (Fig. 6e and f).

## Discussion

In this work, we combined homozygosity mapping, linkage analysis, and WES, to identify the genetic alteration in a consanguineous family with a recessive form of DCM. The identified homozygous mutation in the *FBXO32* gene was uncovered after homozygosity mapping, an approach that we adopted in this case in view of the high probability of identifying a homozygous mutation in a consanguineous family. The parents were asymptomatic with normal echocardiograms, and the disease affected both sexes.

The phenotype in the proband was remarkable, with severe CMP progressing to heart failure that necessitated heart transplantation. Screening family members by echocardiogram revealed variable presentation: two siblings were asymptomatic while the youngest affected child had suggestive cardiac symptoms. This observation underscores the importance of screening first-degree relatives in cases of DCM.

The *FXO32* gene is one of more than 38 members of the FBXO (F-Box Only) family of proteins that have an F-box domain characterized by approximately 50 amino acids, which functions as a site for protein-protein interaction [23]. FBXO32 is one of the components of the E3 ubiquitin ligase SCF (Skp1, Cullin-1, F-box), which binds target proteins for degradation by the UPS. The F-box domain links the F-box protein to the other SCF components by binding Skp1, leading to the destruction of the target proteins. In animals, through enhancing protein degradation, Fbxo32 was initially discovered to play a critical role in inducing muscle atrophy [8, 9].

Serving as a cardiac ubiquitin ligase, *FBXO32* has been implicated in the pathogenesis of CMP.6,7 FBXO32 has been shown to repress calcineurin through assembling with the UPS complex, which promotes cardiac hypertrophy in response to pathologic stimuli [10]. Li et al. have also found that over-expressed Fbxo32 in neonatal rat cardiomyocytes disrupts the Akt-dependent pathway responsible for physiological cardiac hypertrophy.11 Interestingly, down-regulation of Fbxo32 in knockout mice produces the opposite effect, with the inhibition of cardiac hypertrophy in response to pressure overload [24], suggesting that there are different mechanisms by which FBXO32 induces atrophy and hypertrophy in cardiomyocytes [25]. In a recent study on atrogin-1 knockout mice, charged multivesicular body protein 2B (CHMP2B), which is part of an endosomal sorting complex required for autophagy, has been identified as a target of atrogin-1-mediated degradation.12 Mice lacking atrogin-1 fail to degrade CHMP2B, resulting in autophagy impairment, intracellular protein aggregate accumulation, unfolded protein response activation and subsequent cardiomyocyte apoptosis, leading, ultimately, to CMP and premature death.

Furthermore, in support of a potential link between *FBXO32* and DCM in this family, other cardiac ubiquitin ligases (MuRF1, MuRF2, MuRF3, CHIP, MDM2) have also been implicated in the pathogenesis of cardiac hypertrophy, atrophy, and ischemia reperfusion injury.5 Recently, mutations in the cardiac ubiquitin ligase *TRIM63*, which encodes MuRF1, have been identified in patients with familial hypertrophic CMP [26]. The expression of mutant TRIM63 was associated with impaired UPS-mediated protein degradation in cardiomyocytes. This finding, together with our work, suggests that the dysfunction of other proteins in the UPS complexes may also be implicated in the pathogenesis of Mendelian forms of CMP.

Although causality is yet to be established, several factors pertinent to the identified mutation (p.Gly243Arg) support the suggestion that *FBXO32* is a likely candidate gene for DCM. There was a clear co-segregation of the mutation with the clinical phenotype. All of the affected patients, but none of their parents and unaffected siblings, were homozygous for the mutant allele. In addition, the mutation, which replaces a highly-conserved aliphatic non-polar amino acid with a polar positively charged one, was not present in 1986 chromosomes, and was predicted to destabilize the protein.

The identified homoallelic variant in the F-box domain is likely to be a loss-of-function mutation. Being within the F-box domain, this mutation may cause loss of substrate specificity, leading to the premature degradation of functional proteins. It may also cause loss of efficient recruitment of the components of the UPS. However, a gain-of-function mechanism is still a possibility, where the mutant allele could lead to the accumulation of proteins that are damaging. Both mechanisms would theoretically perturb the well-controlled system of protein homeostasis within cardiomyocytes, and lead to CMP.

It is noteworthy that none of the affected patients had skeletal muscle weakness. The mutation identified in this family may have damaged a cardiac-specific protein, and hence the skeletal muscles are spared. However, further studies in cell or animal models harboring this particular mutation may provide a plausible explanation of the mechanism of cardiac disease and of the apparent lack of skeletal involvement.

The methodology that we have adopted in this study has recognizable limitations. The family pedigree was highly suggestive of a recessive pattern of inheritance, and therefore we implemented the approach of homozygosity mapping and WES, followed by excluding non-homozygous variants. Although the recessive inheritance conforms to the segregation of the disease in this family, a dominant pattern with reduced penetrance remains a possibility. In addition, the unaffected individuals may still be in a presymptomatic phase of a late-onset CMP. A long-term follow-up with periodic evaluation may clarify if the identified variant is not fully penetrant. Last, as our study was conducted in a single family, screening *FBXO32* in other families with CMP of unknown etiology may provide more insight and establish the causal relationship between *FBXO32* and CMP.

## Conclusions

Our work suggests that *FBXO32* is a candidate gene for recessive DCM. Further studies are still needed to establish the causal relationship. Genetic analysis for *FBXO32* may be considered in families with recessively inherited DCM of unknown genetic cause.

## Additional file

Additional file 1: Table S1. Forty-eight OMIM genes implicated in dilated and hypertrophic familial cardiomyopathy that were screened for all the homozygous variants detected by whole exome sequencing. All of the identified 62 variants have been previously reported in the dbSNP and/or 1000 Genomes databases as polymorphisms, except for two novel variants in *TNN* and *SGCD* genes, which were found to be intronic. (DOCX 17 kb)
