# Supplementary material for: A substitution mutation in cardiac ubiquitin ligase, FBXO32, is associated with an autosomal recessive form of dilated cardiomyopathy
Source: BMC Med Genet. 2016 Jan 14;17:3. doi: 10.1186/s12881-016-0267-5 (PMC4714499; doi:10.1186/s12881-016-0267-5)
Supplement: Additional file 1: Table S1. — Forty-eight OMIM genes implicated in dilated and hypertrophic familial cardiomyopathy that were screened for all the homozygous variants detected by whole exome sequencing. All of the identified 62 variants have been previously reported in the dbSNP and/or 1000 Genomes databases as polymorphisms, except for two novel variants in TNN and SGCD genes, which were found to be intronic. (DOCX 17 kb) [file 12881_2016_267_MOESM1_ESM.docx]

**Additional file 1: Table S1:** Forty-eight OMIM genes implicated in dilated and hypertrophic familial cardiomyopathy that were screened for all the homozygous variants detected by whole exome sequencing. All of the identified 62 variants have been previously reported in the dbSNP and/or 1000 Genomes databases as polymorphisms, except for two novel variants in *TNN* and *SGCD* genes, which were found to be intronic.

| Gene | Number of all homozygous variants | Number of reported SNPs | Number of novel variants |
| --- | --- | --- | --- |
| *NEXN* | 0 | 0 | 0 |
| *LMNA* | 0 | 0 | 0 |
| *TNNT2* | 0 | 0 | 0 |
| *PSEN2* | 0 | 0 | 0 |
| *ACTN2* | 0 | 0 | 0 |
| *CMYA3* | 0 | 0 | 0 |
| *TTN* | 17 | 16 | 1 (intronic) |
| *DES* | 4 | 4 | 0 |
| *CAV3* | 0 | 0 | 0 |
| *SCN5A* | 1 | 1 | 0 |
| *CMYA1* | 0 | 0 | 0 |
| *MYL3* | 0 | 0 | 0 |
| *TNNC1* | 0 | 0 | 0 |
| *MYOZ2* | 0 | 0 | 0 |
| *SLC25A4* | 0 | 0 | 0 |
| *SDHA* | 0 | 0 | 0 |
| *CMYA5* | 0 | 0 | 0 |
| *SGCD* | 2 | 1 | 1 (intronic) |
| *DSP* | 5 | 5 | 0 |
| *MYO6* | 0 | 0 | 0 |
| *LAMA4* | 4 | 4 | 0 |
| *PLN* | 0 | 0 | 0 |
| *EYA4* | 1 | 1 | 0 |
| *GATAD1* | 0 | 0 | 0 |
| *PRKAG2* | 1 | 1 | 0 |
| *FKTN* | 0 | 0 | 0 |
| *MYPN* | 3 | 3 | 0 |
| *VCL* | 3 | 3 | 0 |
| *LDB3* | 1 | 1 | 0 |
| *RBM20* | 2 | 2 | 0 |
| *BAG3* | 0 | 0 | 0 |
| *CSRP3* | 0 | 0 | 0 |
| *MYBPC3* | 2 | 2 | 0 |
| *CRYAB* | 3 | 3 | 0 |
| *ABCC9* | 3 | 3 | 0 |
| *TMPO* | 0 | 0 | 0 |
| *MYL2* | 0 | 0 | 0 |
| *MYH6* | 1 | 1 | 0 |
| *MYH7* | 3 | 3 | 0 |
| *PSEN1* | 0 | 0 | 0 |
| *ACTC1* | 0 | 0 | 0 |
| *TPM1* | 4 | 4 | 0 |
| *TCAP* | 0 | 0 | 0 |
| *DSG2* | 0 | 0 | 0 |
| *CALR3* | 0 | 0 | 0 |
| *TNNI3* | 1 | 1 | 0 |
| *MYLK2* | 0 | 0 | 0 |
| *JPH2* | 1 | 1 | 0 |
